# Effect of Vitamin Supplementation on Serum Oxidized Low-Density Lipoprotein Levels in Male Subjects with Cardiovascular Disease Risk Factors

**Published:** 2012

**Authors:** Saeid Najafpour Boushehri, Rokiah Mohammad Yusof, Mohammad Nasir Mohammad Taib, Kamran Mirzaei, Narges Yazdekhasti, Samad Akbarzadeh

**Affiliations:** 1 *Faculty of Health, Bushehr University of Medical Sciences. Bushehr, Iran*; 2 *Department of Nutrition and Dietetics. Faculty of Medicine and Health Sciences, University Putra Malaysia, Malaysia*; 3 *Isfahan Cardiovascular Research Center, Isfahan University of Medical Sciences. Isfahan, Iran*

**Keywords:** Cardiovascular Diseases, Oxidized Low-Density Lipoprotein, Vitamin E, Vitamin C, ß-carotene

## Abstract

**Objective(s):**Oxidized low-density lipoproteins (ox-LDLs) appear to play a significant role in atherogenesis. In fact, circulating ox-LDL concentrations have been recognized as a risk factor for cardiovascular disease (CVD). The main objectives of this study were to assess the effects of antioxidant vitamins on ox-LDL as a biomarker of CVD in male subjects with CVD risk factors.

**Materials and Methods:**The effect of antioxidant vitamins on ox-LDL as a biomarker of CVD in male subjects with CVD in male subjects with CVD risk factors at baseline and after 12 weeks of supplementation with vitamin E (400 IU), C (500 mg), ß-carotene (15 mg), and the combined supplements (E, C, and ß-carotene) respectively defined as group E, C, B and control group was considered as group P.

**Results:**The mean values for ox-LDL at the baseline were 86.93 ± 26.30 U/l in group C, 94.52 ± 38.40 U/l in group E, 79.73±2.07 U/l in group B, 85.97±23.07 U/l in combined group, and 84.90± 14.66 U/l in group P. After 12 weeks of intervention the percentage of changes for group C, group E, group B, COM group, and group P were (-18.32), (-2286), (-17.31), (-19.01) and (-2.0), respectively. Using Wilcoxon method, significant differences were detected in the mean ox-LDL concentrations of baseline and after intervention, values in the C, E, B and combined groups (*P*< 005).

**Conclusion:**This study illustrated that diet supplemented with vitamin C (500 mg), vitamin E (400 IU), ß-carotene (15 mg), and the combination of these vitamins was associated with lower serum ox-LDL levels.

## Introduction

Epidemiological studies have shown an inverse relationship between plasma antioxidant vitamins levels (vitamin C and E and ß-carotene) and mortality from coronary heart disease (1). Many studies have reported that the combination of vitamins E and C are more potent in preventing atherosclerosis, and the beneficial effects of vitamin E could be enhanced through the vitamin E regeneration by the assist of vitamin C (2). Oxidized low-density lipoproteins (ox-LDLs) play a substantial role in atherogenesis. In fact, circulating ox-LDL concentrations have been recognized as a risk factor for cardiovascular disease (CVD) **(**3). LDL is an imperative target of oxidation, and oxidative modification of LDL is a key step in the pathogenesis of atherosclerosis. The primary interest in ox-LDL was based on two critical observations. Firstly, ox-LDL was cytotoxic to endothelial and smooth muscle cells (4). Secondly, uptake of native LDL by macrophages occurred at sufficiently low rate to prevent foam cell formation, but uptake of ox-LDL was unregulated and led to macrophage foam cell formation and subsequent atherosclerosis (5). Elevated ox-LDL is independently associated with increased atherosclerotic burden and increased CHD risk (6). It is now clear that ox-LDL, with its many oxidatively modified lipids and degradation products, contributes to the pathophysiology of both the initiation and progression of atherosclerosis (7). Ox-LDL concentrations are strongly correlated with plasma LDL concentrations. The latter is thus a key factor in determining absolute plasma ox-LDL concentration (8). The importance of ox-LDL in atherogenesis has been further confirmed by the use of specific antibodies to oxidize LDL shown to be local to atherosclerotic lesions in the vessel wall (9). It is previously established that oxidative modifications of LDL may result in lipid-induced atherogenesis. All major cells of the arterial wall exert oxidative modifications on LDL particles (10). LDL, which is the major carrier of cholesterol and lipids in the blood (11), intrudes into the intima of lesion-prone arterial sites, where it is oxidized over time by oxidants, generated by local vascular cells or enzymes, to a form with atherogenic properties. There are indirect evidences from *“in vivo”* oxidation studies that have assessed the antioxidant supplementation in animals whose outcomes have supported significant reductions in lesion formation and LDL oxidation through antioxidant supplementation (1). Alpha-tocopherol supplementation in normal volunteers decreased LDL oxidation and its cytotoxic effect on endothelial cells (12). The antioxidants ß-carotene, α-tocopherol, and vitamin C have implicated benevolent effects on blocking or slowing down the atherosclerotic process through the inhibition of LDL oxidation (13). 

## Materials and Methods

This study was carried out at the Persian Gulf Health Research Centre (PGHRC) of Bushehr University of Medical Sciences. Written informed consent was obtained from each participant prior to the study entry and ethical approve was obtained from deputy of research of Bushehr University of Medical Sciences, Iran.

The main objectives of this study were to assess the effects of antioxidant vitamins on biomarkers of CVD in male subjects with CVD risk factors, at baseline and after 12 weeks of supplementation with vitamin E, C, ß-carotene, and the combined supplements. Secondary measurements included anthropometry, biochemical indices and dietary intake that were carried out at baseline and after 12 weeks. Subjects were recruited through advertisement in local newspapers and announcement via radio. Inclusion criteria were age ≥ 40 years, intake of vitamin and mineral supplements during the past 3 months and elevated risk of cardiovascular disease, defined as the presence of the following six known disorders: hypertension, dyslipidemia, type 2 diabetes, obesity, family history of premature CVD, and smoking. Participants with any of the following conditions were excluded from the study: CVD (angina pectoris, myocardial infarction, stroke, peripheral arterial occlusive disease), heart failure, cardiac arrhythmia, type I diabetes, renal insufficiency, active liver disease or cirrhosis and other severe diseases (e.g. cancer, gastrointestinal disorders). People who were unwilling to fulfill the study medications or used out-of-study vitamin supplements were excluded as well. Two hundred individuals who met the inclusion criteria were recruited into the study at baseline and were divided into 5 groups of C, E, B, COM and P. Vitamin supplements were prescribed daily for all the participants in the treatment groups. The group E took vitamin E (400 IU), group C took vitamin C (500 mg), group B took ß-carotene (15 mg), and COM group took vitamin E plus C and ß-carotene by oral supplementation for 12 weeks. Subjects in the control group were requested not to alter their behavior with regards to supplements intake. They were advised to continue with their usual dietary habits and lifestyle. Blood samples were collected after 12 hr of last meal. Fasting venous blood samples were taken for measurement of plasma concentration of ox-LDL, and serum concentrations of vitamin E, C, ß-carotene, FBS, TG, T-Chol, HDL, and LDL cholesterol. Oxidized LDL level was measured using a commercially oxidized LDL ELISA kit (Mercodia Inc, Sweden). The Statistical Package for the Social Sciences (SPSS windows version 15) was applied for statistical analysis. Normality of distribution was evaluated by Kolmogorov-Smirnov test. Since the normality of distribution for biomarkers for CVD was violated, nonparametric statistics like Wilcoxon was employed. For variables that met normality of distribution, Analysis of variance (ANOVA), Independent t test and paired sample t test were utilized to identify the mean differences between and within groups. Statistical significance has been assigned at level of *P*< 0.05.

## Results

A total of 181 out of 200 men completed the study. An overall of 19 people left the study due to personal reasons out of which five were from group C, three from E, six from B and five from P. The mean intakes for calories in the vitamin C, E, beta carotene, combined and control groups were 2517±124 kcal, 2570±209 kcal, 2484 ± 274 kcal, 2506 ± 212 kcal, and 2573±210 kcal, respectively. The major sources of energy were CHO and fat. No significant differences were found in the mean calorie intake and nutrient content of participants’ diet within groups. Table 1 shows the baseline anthropometric measurements of the participants. The mean values of participants’ BMI were 27.86±4.26 kg/m2, 27.28±4.05 kg/m^2^, 26.90±4.66 kg/m^2^, 26.84±3.71 kg/m^2^, and 27.06±3.73 kg/m^2^ in the groups C, E, B, COM and P respectively. At baseline, there were no significant differences (*P*> 0.05) in the mean values anthropometric measurements within the supplementation and control groups.

Table 2 and Table 3 show the mean changes in fasting blood sugar and lipid profile before and after the intervention within groups. In vitamin C group the observed mean changes were as it follows: in FBS -2.51±2.25 mg/dl, TG 38±17.58 mg/dl, TC 6.4±5.31 mg/dl, HDL-C -3.41±1.87 mg/dl and LDL-C 4.60±4.89 mg/dl. However, there were no significant differences (*P*> 0.05) in the mean values of FBS and lipid profile within groups. The mean changes in vitamin E group were -7.91±11.51 mg/dl for FBS, 17.56±14.49 mg/dl for TG, -5.86±3.69 mg/dl for TC, -.961±1.00 mg/dl for HDL-C and -2.06±4.26 mg/dl for LDL-C. The values for group B were respectively 3.177±3.61 mg/dl, 7.91±10.66 mg/dl, -0.02± 3.19 mg/dl, -1.95± 1.25 mg/dl and 6.35± 3.95 mg/dl. Similarly no significant differences were detected in the mean values of FBS, lipid profile within groups E and B. The mean changes at the baseline in the combined (C+E+ ß-carotene) group were as follows: FBS (3.27±3.85 mg/dl), TG (13.35±19.26 mg/dl), TC (4.02±4.36 mg/dl), HDL-C (-0.26±1.35 mg/dl), LDL-C (-2.95±4.35 mg/dl). Placebo (P) group showed the following changes: FBS (-0.65±1.73 mg/dl), TG (6.60±11.17 mg/dl), TC (3.91±2.42 mg/dl), HDL-C (-1.55±1.17 mg/dl), LDL-C (64.40±40.88 mg/dl).

**Table 1:** The anthropometric measurements at the baseline and after the intervention (mean± SD)

	C group (n= 37)	E group (n= 37)	B group (n= 34)	COM group (n= 40)	P group (n= 34)
Parameters					
Height (cm)	172.64±5.78	174.45±6.38	176.85±7.9	175.82±8.8	173.90±7.8
Weight (kg)					
before	83.25±14.69	83.28±14.82	84.19±16.0	83.40±162	82.06±13.5
after	83.36±14.58	83.63±15.35	84.2±16.16	84.38±192	82.13±13.7
*P *value	0.236	0.357	0.540	0.664	0.740
BMI (kg/m2)					
before	27.86±4.26	27.28±4.05	26.90±4.66	26.84±3.1	27.06±3.73
after	27.91±4.27	27.39±4.27	26.91±4.68	27.15±4.92	27.08±3.70
*P* value	0.138	0.396	0.722	0.691	0.679

Note: SD, standard deviation; BMI, body mass index. *P< 0.05

There were also no significant differences in the values of the FBS and lipid profile within groups COM and P (*P*> 0.05).

The initial mean values of ox-LDL were 86.93 ± 26.30 U/l in group C, 94.52 ± 38.40 U/l in group E, 79.73 ± 2.07 U/l in group B, 85.97 ± 23.07 U/l in combined group, and 84.90 ± 14.66 U/l in group P. After 12 weeks of intervention, the mean changes in the ox-LDL level were -15.93± 5.33, -22.56± 5.89, -13.73± 2.9, -16.40± 3.49 and -1.70± 1.31 respectively within C, E, B, COM and P groups. The highest amount of changes belonged to the participants in vitamin E group (-23.86%). Wilcoxon methods showed significant differences in the before and after intervention values of oxLDL concentrations in groups C, E, B, and COM (*P*< 005). No significant changes were observed in the mean values of oxLDL concentrations in the control group.

## Discussion

The current study illustrated that vitamin C (500 mg), vitamin E (400 IU), ß-carotene (15 mg), and the combination of these supplements are associated with reduced serum oxidized LDL (ox-LDL) levels. In more specific, there is a significant difference between vitamins C, E, ß-carotene and the combined groups before and after 12 weeks supplementation.

Strong relationships between antioxidant vitamins (C, E, and ß-carotene) and oxLDL have been reported previously in the literatures. Though the results of current study demonstrated considerable reduction in percentage of the changes of ox-LDL in the intervention groups (17.31- 23.86). The findings of the current study are consistent with those of Reaven *et al* (14), Jialal *et al* (15), and Hodis *et al* (16). 

In a study by Hodis *et al* (16), it was reported that α-tocopherol supplementation (400 IU/day) significantly raised plasma vitamin E levels (*P*< 0.0001), reduced circulating ox-LDL (*P*= 0.03) and reduced LDL oxidative susceptibility (*P*< 0.01). Supplementation with vitamin E (1,600 mg/day) for 3 months has shown a decrease in LDL oxidation by 30-40% (14). The minimum dose of 400 IU/d of α-tocopherol supplementation is needed to significantly reduce the LDL susceptibility to oxidation (15). 

Princen *et al* (17) found that the lag phase of LDL oxidation was significantly increased by as little as 25 mg/day α-tocopherol supplementation. However, it took at least 400 mg/day to show significant reductions in the rate of oxidation. α-Tocopherol supplementation in normal volunteers decreased LDL oxidation and its cytotoxic effect on endothelial cells (12).

**Table 2:** Changes for fasting blood sugar and lipid profiles at the baseline and after the intervention in C and E groups (mean± SD and Mean_d_±SE_d_)

Parameters	C group (n=35)				E group (n=37)			
	Before Mean±SD	After Mean±SD	Mean _d _±SE_ d_ (within groups)	*P *value	Before Mean±SD	After Mean±SD	Mean _d _±SE_ d_ (within groups)	*P *value
FBS (mg/dl)	86.17±24.62	88.28±27.92	-2.51±2.25	0.261	94.29±39.73	102.21±77.20	-7.91±11.51	0.496
TG (mg/dl)	206.65±131.38	168.65±87.16	38±17.58	0.381	241.24±159.66	223.67±129.59	17.56±14.49	0.233
TC (mg/dl)	218.65±36.31	212.25±41.14	6.4±5.31	0.237	208.75±41.31	214.62±45.49	-5.86±3.69	0.121
HDL-C (mg/dl)	45.65±7.95	49.07±12.43	-3.41±1.87	.078	40.18±11.39	41.14±10.36	-.961±1.00	0.345
LDL-C (mg/dl)	132.05±28.32	127.45±29.88	4.60±4.89	.354	124.08±45.59	126.14±40.92	-2.06±4.26	0.631
Vitamin C (µmol/l)	35.15±7.57	48.43±9.95	-13.27±1.26	.000*	35.83±11.62	34.57±9.75	1.26±1.30	0.338

Note: SD = Standard deviation; FBS, fasting blood sugar; TC, total cholesterol; TG, triglycerides; LDL-C, low density lipoprotein cholesterol; HDL-C, high density lipoprotein; mean_d_ = mean of differences; SE_d _= standard error of differences

**Table 3:** Changes for fasting blood sugar and the lipid profiles at the baseline and after the intervention in ß-carotene and combined groups (mean±SD and mean _d_ ±SE)_d_


Parameters	B group (n=34)			COM group (n=40)			P group (n=35)					
	Before Mean±SD	After Mean±SD	Mean _d _±SE _d_ (within groups)	*P *value	Before Mean±SD	After Mean±SD	Mean d ±SE_d_ (within groups)	*P* value	Before Mean±SD	After Mean±SD	Mean_d_ ±SE_d_ (within groups)	*P *value
FBS (mg/dl)	97.02±45.57	91.41±42.08	3.177±3.61	0.386	96.85±37.55	94.72±28.53	3.27±3.85	0.401	83.08±20.543	83.74±21.91	-0.65±1.73	0.708
TG (mg/dl)	220.67±123.28	212.76±116.38	7.91±10.66	0.463	233.75±161.96	220.40±93.42	13.35±19.26	0.492	176.68±91.26	170.08±80.40	6.60±11.17	0.559
TC (mg/dl)	196.85±32.59	197.05±34.22	-0.02±3.19	0.949	209.30±31.92	205.27±44	4.02±4.36	0.363	199.11±36.48	190.91±26.62	3.91±2.42	0.115
HDL-C (mg/dl)	41.32±9.73	43.28±9.68	-1.95±1.25	0.128	42.02±11.78	42.29±10.43	-0.26±1.35	0.847	40.41±8.57	41.96±9.19	-1.55±1.17	0.196
LDL-C (mg/dl)	110.23±29.05	103.88±34.99	6.35±3.95	0.118	118.50±31.36	121.50±28.95	-2.95±4.35	0.502	160.45±24.60	114.05±23.70	64.40±40.88	0.264

Note: SD= Standard deviation; FBS, fasting blood sugar; TC, total cholesterol; TG, triglycerides; LDL-C, low density lipoprotein cholesterol; HDL-C, high density lipoprotein; mean_d_ = mean of differences; SE_d_ = Standard Error of Differences

**Table 4:** Changes in the oxLDL (U/l) in serum at the baseline and after the intervention (mean±SD and mean_d_±SE_d_)

oxLDL (U/l)					
	C group	E group	B group	COM group	P group
Before	86.93± 26.30	94.52 ± 38.40	79.73 ±2.07	85.97±23.07	84.90± 14.661
After	70.99± 16.46	71.95 ± 17.897	65.99±16.72	69.57± 14.99	83.20± 15.68
Mean_d_ ±SE_d _(within groups)	-15.93± 5.33	-22.56± 5.89	-13.73± 2.9	-16.40± 3.49	-1.70± 1.31
Changing Percent	-18.32	-23.86	-17.31	-19.07	-2.0
*P* value	0.005*	0.000*	0.000*	*0.000	0.213

* *P* value <0.05 is significantly different

Test non-parametric, Wilcoxon, was used to compare pre-post tests

In a study by Princen *et al* (17), authors concluded that intake of vitamin E (1,000 IU/day) for 7 days was sufficient to increase plasma alpha-tocopherol and LDL 3.0- and 2.4-folds respectively. Additionally the oxidation resistance of LDL was elevated up to 41%, and the rate of oxidation was lowered by -19% significantly. 

A short study on vitamin C supplementation showed that the exposure of healthy smokers for 4 weeks with 1000 mg ascorbate per day could result in an increase in plasma ascorbate level and a significant reduction in LDL oxidative susceptibility (18).

Supplementation with 400 mg/day vitamin E in normal volunteers has been also effective to lower LDL oxidation resulting in a reduction in its cytotoxic effect on endothelial cells correspondingly (12). In 1996, Omenn *et al* (13) demonstrated that antioxidants beta-carotene, α-tocopherol, and vitamin C are involved with prevention or delay in the atherosclerotic process by inhibiting LDL oxidation.

## Conclusions

The findings of this study demonstrated that supplementation of antioxidant vitamins decreased harmful biomarkers for cardiovascular diseases such as oxLDL. To reduce the risk of CVD occurrence, the individuals at risk should be encouraged for a daily consumption of antioxidant vitamins supplementation.
